# Factors Affecting the Patterns of Total Amount and Proportions of Leukocytes in Bovine Milk

**DOI:** 10.3390/ani10060992

**Published:** 2020-06-06

**Authors:** Alfonso Zecconi, Lucio Zanini, Micaela Cipolla, Bruno Stefanon

**Affiliations:** 1 Department of Biomedical, Surgical and Dental Sciences-One Health Unit, University of Milan, Via Pascal 36, 20133 Milano, Italy; 2 Associazione Regionale Allevatori Lombardia, Via Kennedy 30, 26013 Crema, Italy; l.zanini@aral.lom.it (L.Z.); m.cipolla.cmh@gmail.com (M.C.); 3 Department of Agricultural and Environmental Sciences, University of Udine, Via delle Scienze 206, I-33100 Udine, Italy; bruno.stefanon@uniud.it

**Keywords:** differential cell count, somatic cell, immune response, milk leukocytes

## Abstract

**Simple Summary:**

Differential leukocyte count (DSCC) in milk is considered important to improve our knowledge on udder immune response since it describes the proportions of leukocytes in milk. However, we hypothesized that the total amount of each cell population in daily milk production would be even more useful. Therefore, we analyzed the pattern of both DSCC and the total amount of polymorphonuclear neutrophils (PMN) + lymphocytes (LYM) (P + LT), calculated as SCC × milk yield × DSCC (as proportion). Cows with ≤200,000 cells/mL have a P + LT average between 5.0 × 10^8^ and 3.0 × 10^9^ cells. In cows with SCC >200,000 cells/mL, the values were 1.6 × 10^10^ and 2.5 × 10^10^ cells. Therefore, the presence of a well-defined inflammatory process increased the overall amount of PMN and lymphocytes LYM of 1 log, from 1 × 10^9^ to 1 × 10^10^. The assessment of the total amount of PMN and LYM, to our knowledge, has never been reported in scientific literature and the value reported may be proposed as benchmarks for studies on udder immune response. The results of this study showed that cows in first and second lactation have a significant lower amount of PMN + LYM, when compared to cows in third and higher lactation. However, these differences are numerically not very large (7%), suggesting that, in healthy animals, the number of immune cells is kept as constant as possible. To the best of our knowledge, this is the first study describing the pattern of DSCC and the total amount of PMN + LYM in relation to parity, days in milk, and SCC, and it may be considered as a first contribution in the investigation on mammary gland immune response by means of differential cell counts in milk.

**Abstract:**

Differential leukocyte count (DSCC) in milk is considered important to improve knowledge of udder immune response. The investigations on milk DSCC were limited by the techniques available until recently, when a high-throughput tool to perform DSCC opened the way to explore these factors in rapid and economically sustainable ways. We hypothesized that DSCC alone does not fully describe the pattern of these cells, since the total amount is also influenced by milk yield and SCC. Therefore, this study was designed to describe DSCC and total amount of different leukocytes in milk during the course of lactation in cows differing in parity and in levels of SCC. This study considered 17,939 individual milk tests from 12 dairy herds in Lombardy Region, where DCC testing was applied in the period of February 2018–December 2019 (23 months). The samples were divided into two subsets—“healthy” (HS) with SCC ≤200,000 cells/mL and “inflamed” (IS) with SCC >200,000 cells/mL. Cow in HS have a P + LT average between 5.0 × 10^8^ and 3.0 × 10^9^ cells. In IS cows, the values were 1.6 × 10^10^ and 2.5 × 10^10^. Therefore, the presence of a well-defined inflammatory process increased the overall amount of polymorphonuclear neutrophils (PMN) and lymphocytes (LYM) of 1 log, from 1 × 10^9^ to 1 × 10^10^. The assessment of the total amount of PMN and LYM, to our knowledge, have never been reported in scientific literature; the values observed may be proposed as benchmarks for studies on udder immune response. When data were analyzed by days in milk (DIM), they showed that cows in first and second lactation have a significantly lower amount of PMN + LYM, when compared to cows in third and higher lactation. However, these differences are numerically not very large (7%), and suggest that, in healthy animals, the number of immune cells is kept as constant as possible. In IS, the analysis of trends based on DIM showed that both DSCC and P + LT have a significant negative trend. These data suggest that only in this group, the presence of high SCC as lactation proceeds is associated with a progressive increase in the number of macrophages. To the best of our knowledge, this is the first study describing the pattern of DSCC and the total amount of PMN + LYM in relation to parity, days in milk, and SCC, and it may be considered as the first contribution in the investigation on mammary gland immune response by the means of differential cell counts in milk.

## 1. Introduction

The role of leukocytes in the defense of the mammary gland is well known [1,2], and differential leukocyte count (DSCC) is considered important information on udder immune response and to improve mastitis diagnosis [3,4,5,6]. Indeed, somatic cell counts (SCC), the tool currently applied for this aim, is not able to completely describe the defense mechanisms within the udder [7]. Until recently, investigations on milk DSCC were limited by the available investigation techniques: direct microscopical and flow-cytometry analysis. Both these techniques have poor reproducibility, high costs, and are labor-intensive, thus limiting their application to studies with a relatively small sample size. These technical aspects and small sample sizes, in addition to different designs of experiments and sampling procedures could explain why there are very large and overlapping ranges among studies on leukocyte proportions [4,7,8,9,10,11]. Moreover, the problems previously mentioned reduce the chance to analyze DSCC in cows with different physiological, pathological, and lactation stages. Indeed, SCC vary in relation to parity, days in milk (DIM), health, and welfare status [12], and these factors very likely have an influence on DSCC.

The recent availability of a high-throughput tool to perform DSCC opens the way to explore these factors in an economically sustainable way, allowing proper sample sizes and compositions [13]. This new tool allows to identify within a milk sample the macrophages (MAC) and the combination of polymorphonuclear neutrophils (PMN) and lymphocytes (LYM). Diagnostic characteristics and performances were described by Damm et al., 2017 [13]. DSCC is expressed as the combined proportion (%) of PMN and LYM on the overall count of milk cells.

To the best of our knowledge, studies on leukocyte patterns throughout lactation and parities are scarce and the few available are focused on specific aspects, such as the association with SCC or the values at the end of lactation [14,15,16,17]. Moreover, studies on the variability of the total amount of different leukocytes in milk are scarcer [4,18], and based on a very small sample size. We hypothesized that DSCC alone do not fully describe the pattern of leukocytes in milk.

This study was designed to describe DSCC and total amount of different cells in milk in cows during the course of lactation with different parity and levels of SCC.

## 2. Materials and Methods

### 2.1. Herd and Cow Selection

This study considered 17,939 milk test records from 12 dairy herds in Lombardy Region where DCC testing was applied for the period of February 2018–December 2019 (23 months).

### 2.2. Sample Collection

Individual cow samplings were performed by certified methods currently applied by Italian Breeders Association at the laboratories of Regional Breeders Association of Lombardy (ARAL) by the means of Lactocorder™ (WMB AG, Balgach, Switzerland). Samples were taken about every 5 weeks, delivered refrigerated to ARAL labs the same day, and analyzed within 30 h from sampling.

### 2.3. Milk Composition Analysis

Milk analyses included SCC and DSCC and were carried out on Fossomatic™ 7DC (Foss A/S, Hillerød, DK). The analysis of DSCC was based on Foss DSCC Method Cell Staining (international patent PCT/EP2010/065615–Holm, 2012), as described by Damm et al. [13]. The method allows to identify within a milk sample the macrophages (MAC) and the combination of PMN and LYM. Diagnostic characteristics and performances were described by Damm et al. [13]. DSCC is expressed as the combined proportion (%) of PMN and LYM on the overall count of milk cells.

### 2.4. Cow and Milk Test Record Data

Only cows that had a minimum of 2 and a maximum of 12 valid individual milk samples (IMS) were included in the study. Individual milk samples’ data and cow data, recorded by ARAL, included: herdID, cowID, parity (n), days in milk (d), milk yield (kg), SCC (cells/mL), D**S**CC (%). Cow data and milk test data were combined in a database for statistical analyses.

To assess the pattern of the total amount of PMN and LYM included in the milk, a new variable was calculated. Total PMN + LYM (P + LT) is equal to SCC × milk yield × DSCC (as proportion). This variable is expressed as log_10_ of the value obtained from the previous formula and represents the total amount of PMN and LYM in the milk produced on the day of sampling.

### 2.5. Datasets for Analyses

The database was split into two subsets, one including samples up to 200,000 cells/mL (Healthy set–HS) and the other one with samples >200,000 cells/mL (Inflamed set–IS). Healthy subset were furthermore ranked by SCC thresholds usually applied in field conditions: 100,000 cells/mL [19]; 200,000 cells/mL [20,21,22] when SCC are very low (≤50,000 cells/mL) and at an intermediate level (150,000 cells/mL). Usually, cows below these thresholds are considered healthy, even if the presence of an IMI cannot be excluded [16]. Cows with SCC ≥ 200,000 cells/mL (IS) are considered with subclinical mastitis, and in this group, we also considered higher SCC levels (400,000 and 800,000 cells/mL).

The rationale for the classification of HS is to describe the DSCC and P + LT pattern starting with very low SCC cows (≤50,000 cells/mL), which have a very low chance of an inflammatory process, and, progressively, exploring patterns in cows with increasing SCC levels. Similarly, groups in IS were defined to describe DSCC and P + LT patterns in cows having an increasing level of inflammation, as suggested by the increase in SCC [23,24].

### 2.6. Statistical Analysis

Data were analyzed by the Mann-Kendall test to identify trends during lactation. This analysis allows to identify not only the presence of a trend in the overall series, but also if there is a trend from one month to another [25]. This analysis was performed by means of XLSTAT 2020.1.3 software (Addinsoft, New York, NY, USA).

An analysis for repeated measures (HP MIXED procedure; SAS Institute Inc., Cary, NC, USA) was used to assess the association of DSCC and P + LT with parity, days in milk (DIM), and SCC as a fixed effect. Herds and cows were included in the model as random effects.

The statistical model, for both DSCC and P + LT, was the following:

Y_mnopq_ = μ + Herd_m_ + Cow_n_ + Parity_o_ + DIM_p_ + SCC_q_ + SCC_q_ × Parity_o_ + SCC_q_ × DIM_p_ + e_mnopq_;

where Y_mnopq_ is the observed value for DSCC or P + LT; μ is the overall mean; Herd_m_ is the random effect of the m^th^ herd (m = 1 to 12); Cow_n_ is the random effect of the n^th^ animal (p = 1 to 2425); Parity_o_ is the fixed effect of the o^th^ class of parity (o = 1 to 4); DIM_p_ is the fixed effect of the p^th^ class of days in milk (n = 1 to 13); SCC_q_ is the fixed effect of the q^th^ class of SCS (q = 1 to 4 in HS and 1 to 3 in DS); SCC_r_ × Parity_s_ is the fixed effect of the interaction between the q^th^ class of SCC and the o^th^ class of Parity; SCC_q_ × DIM_p_ is the fixed effect of the interaction between the q^th^ class of SCC and the p^th^ class of DIM; e_mnopqi_ is the residual error.

## 3. Results

### 3.1. Data Description

The study included 2425 cows from 12 different herds, and sample proportions among herds were in the range of 4.8–24.8%. Italian Holstein Frisian was the breed with highest frequency (96.5%); most of the remaining ones were Italian Brown Swiss (3%), while other breeds or cross breeds represented 0.5% of the sample. The description of the main characteristics of the dataset is given in Table 1, while individual herd data are reported in Supplementary Table S1.

Overall, 17,939 individual milk samples were considered for the statistical analysis, fulfilling the requirements described in the Material and Method section. Cows in first lactation made up 40.2% of the total, cows in second lactation represented 27.6% of the samples, 17.2% were in third lactation, and the proportion of older cows was 15.0%. The distribution of samples among DIM is reported in Supplementary Figure S1.

The average milk yield was 35.2 ± 11.8 Kg/d with a range 27–47 Kg/d (Table S1), while SCC mean was 5.0 ± 4.9 log_10_ cells/mL and DSCC mean was 62.0 ± 17.3%. The total amount of P+LT was 9.3 ± 0.7 log_10_ cells with a range between 8.8 and 9.1 log_10_ cells.

The main characteristics of the two subsets (HS and IS) were reported in Supplementary Table S2. As expected, the frequency of cows in HS increased with increasing SCC, whereas the frequency decreased in IS as SCC increased.

### 3.2. Factors Affecting Differential Somatic Cells Patterns

In Table 2 the results of statistical analyses performed on the two datasets were reported, HS and IS, to evaluate the influence of the random and fixed factors on the variance of DSCC and P + LT. Among random factors, cows showed a larger influence than herds on both DSCC and P + LT. The influence of cows was smaller in HS than in IS and, in the latter, it was greater for DSCC than for P + LT. Herds play a smaller role than cows on the variance of DSCC and P + LT in IS, and it was even lower in HS for DSCC.

With the fixed factors considered, their interactions showed a significant influence on the variance of both DSCC and P + LT in HS. In IS, only parity and DIM showed statistically significant effects on both cells’ measures, while interactions had no influence on the variance of DSCC.

The distribution of mean values for the fixed factors (SCC and parity) in HS is given in Table 3. DSCC mean values were statistically different when ranked by SCC. As expected, samples with ≤50,000 cell/mL had the lowest DSCC mean (50%), and this value significantly increased in higher SCC classes. When the same data were ranked by parity, primiparous cows showed significantly higher DSCC values when compared to older cows. The mean value drops in second parity cows of about 6%, to increase again as parity increases.

The same analysis applied to P + LT values (Table 3) revealed a similar scenario with a lower total number of PMN and LYM in primiparous cows, and a significant increase as SCC levels increase. Conversely, when parities were considered, cows in the first two lactations had a lower total number of PMN and LYM when compared to older cows, with differences in a range between 6.5 × 10^7^ and 1.6 × 10^8^ cells.

The data obtained from the analysis of interactions between SCC and parity confirmed that primiparous cows has significant higher DSCC values, irrespective of SCC levels (Table 4). These values dropped in cows in second lactation, to marginally increase again as parity increases; significant differences were not observed in samples ≤150,000 cells/mL and ≤200,000 cells/mL. SCC classes were always associated with significant differences in DSCC for all the parities considered.

The same analysis applied to P + LT data (Table 5) showed that cows with ≤50,000 cell/mL had a significant lower number of PMN and LYM when compared to the other SCC classes. Despite the statistically significant differences, it should be noted that the difference between the lowest value observed (primiparous cows with ≤50,000 cell/mL) and the highest (older cows with ≤200,000 cells/mL) is in the range of 4.4–6.9%. An increasing trend in P + LT values was observed both when SCC and parity number increased, but values in cows with ≤200,000 cells/mL were not statistically different among parities.

When the inflamed subset was considered, only fixed factors showed a significant influence on DSCC and P + LT variability (Table 2). DSCC mean values as well as P + LT values were considerably higher in this subset when compared to HS as well as P + LT. Primiparous cows were confirmed to have numerically higher DSCC values than older cows, but the statistically significant differences were not consistent. The opposite pattern was observed for P + LT, and in this case, the statistically significant differences were not consistent (Table 6).

### 3.3. Cellular Pattern during Lactation.

**Figure 1:**
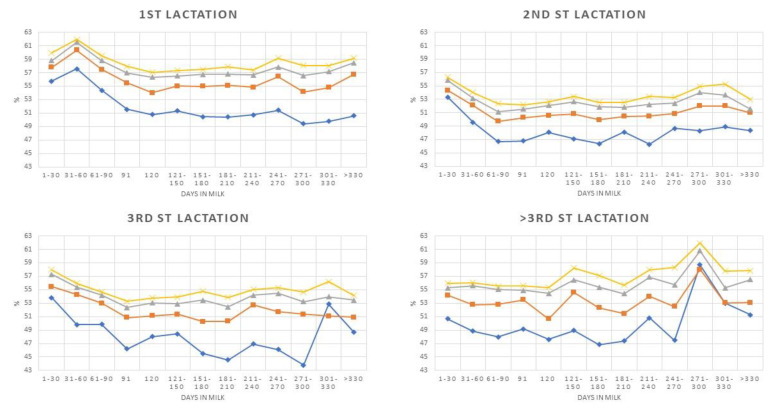
Differential Somatic Cell Count patterns during lactation classified by parity and Somatic Cell Count level (♦ ≤50,000 cells/mL; ■ ≤100,000 cells/m; 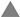 ≤150,000 cells/m; 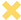
≤200,000 cells/m).

**Figure 2:**
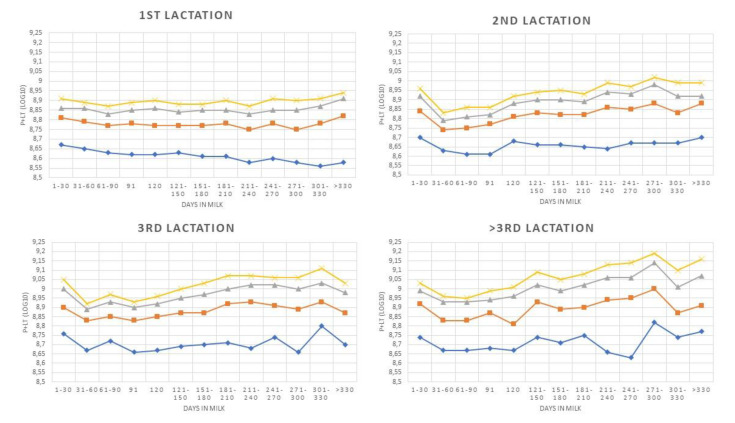
P + LT patterns during lactation classified by parity and Somatic Cell Count level (♦ ≤50,000 cells/mL; ■ ≤100,000 cells/m; 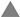 ≤150,000 cells/m; 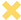
≤200,000 cells/m). P+LT is equal to SCC × milk yield × DSCC (as proportion).

**Figure 3:**
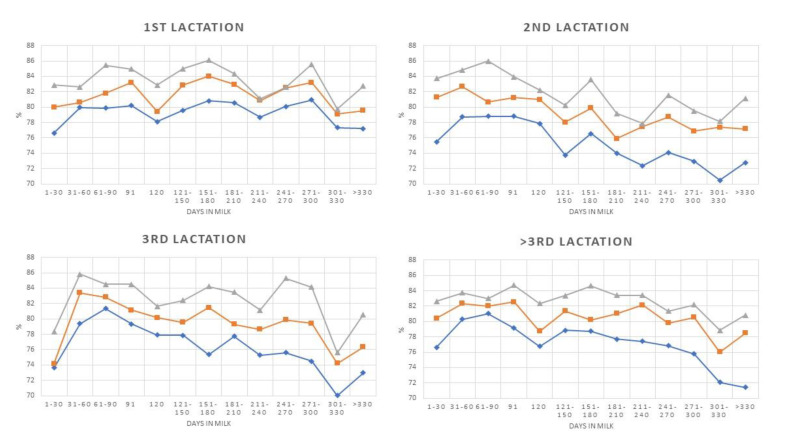
Differential Somatic Cell Count (DSCC) patterns during lactation classified by parity and Somatic Cell Count (SCC) level (♦ >200,000 cells/mL; ■ >400,000 cells/m; 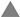 >800,000 cells/mL.

**Figure 4:**
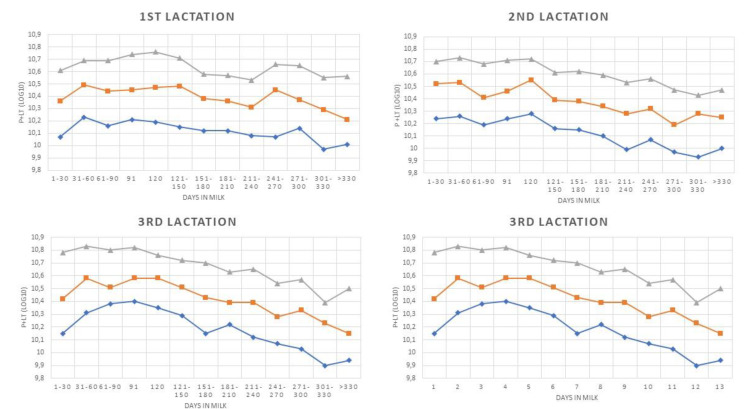
PL + T patterns during lactation classified by parity and Somatic Cell Count level (>200,000 cells/mL; >400,000 cells/m; >800,000 cells/m). P + LT is equal to SCC × milk yield × DSCC (as proportion).

The presence of a consistent significant influence of DIM on DSCC and P+LT variance and our interest in assessing the pattern of cellular immune response suggested analysis of these aspects in depth. Figure 1, Figure 2, Figure 3 and Figure 4 report the pattern of DSCC and P + LT based on Parity and SCC levels for both healthy and inflamed subsets. The DSCC pattern in HS showed that the mean values in samples ≤50,000 cells/mL were always largely below the means observed for samples with higher SCC, with very few exceptions at the end of lactation in cows with three or more parities (Figure 1). An increase of DSCC mean values can be observed as SCC levels increased, as expected. The analysis of the curves reported in Figure 1 did not show any trend, except for cows ≤50,000 cells/mL with 1 and 3 parturitions, where a significant negative trend was observed (Table 7). Conversely, a significant positive trend was observed for older cows with SCC ≤200,000 cells/mL.

These patterns were only partially confirmed by analysis of P + LT values (Figure 2). Indeed, trend analysis showed a significant positive trend for most of the curves. The few exceptions are represented by cows with more than one parturition with ≤50,000 cells/mL, where the trend observed was not significant, whereas a significant negative trend was observed for primiparous cows ≤50,000 cells/mL.

The same analytical approach was applied to IS, and the results showed, as expected, higher DSCC and P + LT mean values when compared with HS. A significant negative trend was observed for all DSCC curves out of those concerning primiparous cows. Mean DSCC values were higher as SCC threshold increased, as observed in HS. The analysis of the P+LT pattern showed that all the trends were negative (*p* < 0.05), and P + LT mean values increased with increasing SCC levels, as expected.

## 4. Discussion

In this study, herd selection was not random, constrained by the necessity to have DSCC data over at least a 12 months-period; however, herd size, milk yield, and SCC are similar to the ones observed in previous larger studies on the same area [16,24,26].

As expected, primiparous cows were the prevalent group among parities (40%), as well as 73% samples were with ≤200,000 cells/mL. Samples with ≤50,000 cells/mL had a frequency of 39%, confirming the high prevalence of cows with very low SCC [16,26].

To the best of our knowledge, this is the first time that the total number of cells was considered to analyze leukocytes pattern in milk. Our data showed that the milk collected during a whole milking contains an average of 1 × 10^9^ PMN and LYM. Cows in HS have a P + LT average between 5.0 × 10^8^ and 3.0 × 10^9^ cells (Table 3), while in the IS group, the values were in the range of 1.6 × 10^10^ and 2.5 × 10^10^ cells (Table 6).

Despite the applied technique not being able to differentiate PMN from LYM, previous data suggested that PMN is the predominant cell type when SCC is largely below ≤100,000 cells/mL [27], and that LYM is in the range of 1%–19%, independent of the SCC of the samples [13]. Therefore, the changes in DSCC and in P+LT in relation to SCC levels should high likely be related to variation in PMN concentrations.

The presence of a well-defined inflammatory process (samples in IS subsets) increased the overall amount of PMN and LYM of 1 log, from 1 × 10^9^ to 1 × 10^10^ or, in other words, of about 1 billion cells, most of which are presumably PMN. This change is the result mainly of overall increase in SCC, rather than changes in DSCC proportions. Indeed, in the latter case, the increase is in the range of 15–25%, when DSCC proportions were compared in samples with the lowest SCC (≤50,000 cells/mL) and the highest SCC (>800,000 cells/mL). The assessment of total amount of PMN and LYM, to our knowledge, has never been reported in scientific literature; the value reported may be proposed as a benchmark for studies on immune response in the mammary gland.

The availability of a sample size larger than the ones reported even in recent studies on DSCC [14,15,16,24,28] and the period of time during which samples were collected allows to investigate the pattern of cell types during the course of the whole lactation. The statistical model applied for evaluating factors affecting DSCC variability in a healthy subset showed that, among random factors, the herd has a very low influence, while all fixed factors considered, as well as their interactions showed a statistically significant influence (Table 2). These results were consistent among the four different SCC thresholds considered, similar to what was observed in studies on SCC [12]. The increase in SCC levels were associated with an increase in DSCC as expected, but our data showed a decrease of DSCC values as parity increased (Table 4). The trend analysis showed a decrease of DSCC with increasing DIM when SCC was ≤50,000 cells/mL, but no significant trends were observed at higher SCC levels (Table 7). These data did not agree with the results of studies applying flow-cytometry [29], neither with the ones from a recent study applying the same instrument as in this study [14]. However, in the latter case, data were not ranked by SCC levels.

When P + LT were considered, a different scenario appears. Indeed, cows in first and second lactation have a significant lower amount of PMN and LYM, when compared to cows in third and higher lactation (Table 3). However, these differences are numerically not very large (7%), and they suggest that, in healthy animals, the number of immune cells is kept as constant as possible, as previously suggested [30], and a reference value of 1.4 × 10^9^ for P + LT in healthy cows can be proposed. Therefore, primiparous cows cannot be considered to have a different level of cell immunity compared to older cows, as suggested by the higher DSCC values observed. Indeed, the DSCC peak observed at the beginning of lactation in cows smoothen when P + LT is considered (Figure 1 and Figure 2). Very likely, the higher proportion of PMN and LYM are needed to compensate both the lower SCC levels and yield, generally observed in primiparous cows, in order to keep PMN and LYM at the proper level. These data also confirm that cows with very low SCC are not at greater risk of developing mastitis [31], since there are not large differences in total milk PMN + LYM, when compared to older cows.

Often dilution or concentration effects are suggested as explanation of abnormal changes in SCC in healthy cows [32]. The data of our study did not support this hypothesis in healthy cows. Indeed, trends are generally positive for P + LT as DIM increases, suggesting that the overall amount of PMN and LYM released into milk increases when milk yield generally decreases, irrespective of the number of lactations. These increases may be related to the prolonged exposure to bacteria during lactation, thus stimulating epithelial cells, which release pro-inflammatory mediators [33,34] and increasing the recruitment of leukocytes from blood circulation.

The same analysis applied to the cows with >200,000 cells/mL (IS) showed that the role of herd, as random factor, has proportionally a relatively higher influence on DSCC and P + LT variance, in comparison with HS (Table 2). These data suggest that the level of inflammation, herd management and, very likely, bacteria prevalence play an important role in modulating cellular immune response in diseased animals. These observations are supported by the absence of an influence from the interaction between SCC and parity or DIM.

The overall mean values for both DSCC and P + LT in IS are largely higher than the values in HS (Table 6 and Figure 3 and Figure 4). An increase in DSCC with rise in SCC was also observed in this group, but the influence of parity on DSCC was less consistent. Significant differences were observed, but without a clear trend, while P + LT showed patterns similar to the ones observed in HS with a large increase in cell concentration from third parity and over (Table 6).

The analysis of trends based on DIM showed that both DSCC and P + LT show a significant negative trend (Table 7), with a few exceptions for DSCC (primiparous cows and samples >800,000 cells/mL). These data suggest that in this group, the presence of high SCC as lactation proceeds is associated with a progressive increase in the number of macrophages. An increase in the proportion of macrophages in late lactations have been reported in previous studies on quarter milk samples [17,35], but these studies involved both healthy cows and with subclinical mastitis. In our study, reduction in the proportion and correspondent increase in overall number of macrophages was observed only in cows with a clear inflammatory process; these results may be related to the increase frequency of chronic mastitis in late lactation [36,37].

## 5. Conclusions

To the best of our knowledge, this is the first study describing the DSCC pattern and the total amount of PMN + LYM in relation to parity, days in milk, and SCC levels. The results of this study suggest that the total number of PMN + LYM is kept as stable as possible during lactation and among parities in healthy animals. This is obtained by modulating the proportion of these cells in relation to milk yield and the SCC, leading to changes in DSCC, unrelated to the presence of a disease. A healthy cow showed to have about 1 × 10^9^ PMN + LYM in milk produced in a single day. The values obtained from this study may be proposed as benchmarks for further studies on immune response of the mammary gland.

## Figures and Tables

**Table 1:** Description of the main characteristics of the dataset analyzed (17,939 individual milk samples from 2425 cows).

Parameter	Units	Mean	Median	Std Dev ^4^	Minimum	Maximum
Parity	Number of parturitions	2.15	2.0	1.28	1	9
Days in milk	days	173.6	165.0	106.30	5	420
Milk yield	Kg/d	35.15	33.6	11.83	3.9	91.5
SCC ^1^	(log_10_SCC/mL)	4.97	4.86	0.63	3.00	7.55
DSCC ^2^	%	61.97	63.3	17.26	1.50	97.10
P + LT ^3^	Log_10_	9.27	9.2	0.69	6.97	11.85

^1^ SCC = Somatic Cell Count. ^2^ DSCC = Differential leukocyte count. ^3^ P + LT is equal to SCC × milk yield × DSCC (as proportion). ^4^ Std Dev = Standard deviation.

**Table 2:** Results of ANOVA analysis for repeated measures applied to the two subsets considered (healthy and inflamed).

Dataset	Response Variable	Cow	Herd	Parity	DIM	SCC	SCC × Parity	SCC × DIM
Healthy	DSCC ^1^	31.8%	0.07%	<0.0001	<0.0001	<0.0001	<0.0001	0.0006
	P + LT ^2^	32.7%	2.2%	<0.0001	<0.0001	<0.0001	<0.0001	<0.0001
Inflamed	DSCC	54.4%	6.1%	<0.0001	<0.0001	<0.0001	n.s.	n.s.
	P + LT	46.1%	3.7%	<0.0001	<0.0001	n.s.	n.s.	n.s.

^1^ DSCC = Differential leukocyte count. ^2^ P + LT is equal to SCC × milk yield × DSCC (as proportion). n.s. = not significant.

**Table 3:** DSCC and P + LT mean and standard error in healthy subset ranked by SCC levels and parities.

SCC^1^ (cells/mL)	DSCC ^2^	P + LT ^3^	Parity	DSCC	P + LT
≤50,000	50.22 ^a^ ± 0.41	8.67 ^a^ ± 0.01	1	63.82 ^a^ ± 0.44	9.14 ^a^ ± 0.01
≤100,000	59.37 ^b^ ± 0.41	9.10 ^b^ ± 0.01	2	57.79 ^b^ ± 0.45	9.13 ^a^ ± 0.01
≤150,000	64.20 ^c^ ± 0.45	9.35 ^b^ ± 0.01	3	58.59 ^c^ ± 0.49	9.16 ^b^ ± 0.01
≤200,000	65.77 ^d^ ± 0.56	9.49 ^d^ ± 0.01	≥4	59.35 ^c^ ± 0.55	9.18 ^c^ ± 0.01

^1^ SCC = Somatic Cell Count. ^2^ DSCC = Differential leukocyte count. ^3^ P + LT is equal to SCC × milk yield × DSCC (as proportion). Rows with different superscripts (^a,b,c,d^) are statistically different (*p* < 0.05)

**Table 4:** Differential leukocyte count (DSCC) mean and standard error in healthy subset ranked by Somatic Cell Count (SCC) levels.

SCC Level (cells/mL)	≤50,000		≤100,000		≤150,000		≤200,000	
Parity	Mean	Std.err ^1^	Mean	Std.err	Mean	Std.err	Mean	Std.err
1	53.71 ^a^	0.41	63.60 ^a^	0.43	67.78 ^a^	0.52	70.22 ^a^	0.77
2	48.65 ^b^	0.42	56.96 ^b^	0.44	61.52 ^b^	0.57	63.64 ^b^	0.82
3	49.28 ^c^	0.47	58.33 ^c^	0.49	63.14 ^b^	0.64	63.61 ^b^	0.96
≥4	49.24 ^c^	0.54	58.60 ^c^	0.56	63.96 ^b^	0.70	65.62 ^b^	1.03

^1^ Std.err = Standard error of the mean. Rows with different superscripts (^a,b,c,d^) are statistically different (*p* < 0.05).

**Table 5:** P + LT ^1^ mean and standard error in healthy subset ranked by SCC levels.

SCC Level (cells/mL)	≤50,000		≤100,000		≤150,000		≤200,000	
Parity	Mean	Std.err ^2^	Mean	Std.err	Mean	Std.err	Mean	Std.err
1	8.64 ^a^	0.01	9.09 ^a^	0.01	9.34 ^a^	0.01	9.51 ^a^	0.02
2	8.65 ^b^	0.01	9.08 ^a^	0.01	9.32 ^a^	0.01	9.47 ^a^	0.02
3	8.69 ^c^	0.01	9.10 ^b^	0.01	9.36 ^b^	0.01	9.49 ^a^	0.02
≥4	8.70 ^c^	0.01	9.13 ^c^	0.01	9.38 ^c^	0.02	9.51 ^a^	0.02

^1^ P + LT is equal to SCC × milk yield × DSCC (as proportion). ^2^ Std.err = Standard error of the mean. Rows with different superscripts (^a,b,c,d^) are statistically different (*p* < 0.05).

**Table 6:** DSCC and P + LT mean and standard error in diseased subset ranked by SCC levels and parities.

SCC ^1^ (cells/mL)	DSCC ^2^	P + LT ^3^	Parity	DSCC	P + L Total
>200,000	75.60 ^a^ ± 0.95	10.17 ^a^ ± 0.03	1	78.26 ^a^ ± 0.99	10.16 ^a^ ± 0.03
>400,000	76.87 ^b^ ± 0.96	10.28 ^a^ ± 0.03	2	75.65 ^b^ ± 0.98	10.18 ^a^ ± 0.03
>800,000	78.48 ^c^ ± 0.97	10.43 ^a^ ± 0.3	3	75.61 ^c,d^ ± 1.00	10.85 ^b^ ± 0.03
			≥4	77.41 ^a,d^ ± 1.02	10.28 ^c^ ± 0.03

^1^ SCC = Somatic Cell Count. ^2^ DSCC = Differential leukocyte count. ^3^ P + LT is equal to SCC × milk yield × DSCC (as proportion). Rows with different superscripts (^a,b,c,d^) are statistically different (*p* < 0.05)

**Table 7:** Results of the Mann-Kendall test to identify trends during lactation for Differential Somatic Cell Count (DSCC) and P + LT ranked by Somatic Cell Count (SCC) levels and parity in healthy and diseases subsets.

	DSCC				P + LT ^1^			
Parity SCC Levels (cells/mL)	1	2	3	>3	1	2	3	>3
≤50,000	−0.090 *	−0.032	−0.080 *	0.013	−0.059 *	0.028	0.012	0.018
≤100,000	−0.026	0.001	−0.033	0.023	0.020	0.096 *	0.077 *	0.085 *
≤150,000	0.009	0.007	−0.016	0.044	0.042 *	0.107 *	0.089 *	0.107 *
≤200,000	0.000	0.014	−0.006	0.066 *	0.048 *	0.111 *	0.098 *	0.134 *
>200,000	−0.027	−0.119 *	−0.111 *	−0.101 *	−0.074 *	−0.147 *	−0.154 *	−0.130 *
>400,000	−0.042	−0.116 *	−0.108 *	−0.057 *	−0.107 *	−0.159 *	−0.157 *	−0.132 *
>800,000	−0.036	−0.120 *	−0.079	−0.049	−0.121 *	−0.144 *	−0.210 *	−0.160 *

^1^ P + LT is equal to SCC × milk yield × DSCC (as proportion). * trend statistically significant (α = 0.05).

## Supplementary Materials

The following are available online at https://www.mdpi.com/2076-2615/10/6/992/s1, Figure S1: Distribution of samples by days in milk, Table S1: Main characteristics of the 12 herds included in the study, Table S2 Distribution of samples within the two data subsets (healthy and diseased) ranked by parity and SCC.

## References

[1] Paape M.J., Mehrzad J., Zhao X., Deteilleux J., Burvenich C. (2002). Defense of the bovine mammary gland by polymorphonuclear neutrophil leukocytes. J. Mammary Gland. Biol. Neoplasia.

[2] Zecconi A., Smith K.L. (2003). Ruminant Mammary Gland Immunity.

[3] Pillai S.R., Kunze E., Sordillo L.M., Jayarao B.M. (2001). Application of differential inflammatory cell count as a tool to monitor udder health. J. Dairy Sci..

[4] Rivas A.L., Quimby F.W., Blue J., Coksaygan O. (2001). Longitudinal evaluation of bovine mammary gland health status by somatic cell counting, flow cytometry, and cytology. J. Vet. Diagn. Investig..

[5] Vangroenweghe F., Dosogne H., Burvenich C. (2002). Composition and milk cell characteristics in quarter milk fractions of dairy cows with low cell count. Vet. J..

[6] Mehrzad J., Paape M., Burvenich C. (2010). Role of neutrophils in protection of udder from infection in high yielding dairy cows. Iran J. Vet. Res..

[7] Merle R., Schroder A., Hamann J. (2007). Cell function in the bovine mammary gland: A preliminary study on interdependence of healthy and infected udder quarters. J. Dairy Res..

[8] Koess C., Hamann J. (2008). Detection of mastitis in the bovine mammary gland by flow cytometry at early stages. J. Dairy Res..

[9] Leitner G., Eligulashvily R., Krifucks O., Perl S., Saran A. (2003). Immune cell differentiation in mammary gland tissues and milk of cows chronically infected with Staphylococcus aureus. J. Vet. Med. Ser. B.

[10] Schwarz D., Diesterbeck U.S., Konig S., Brugemann K., Schlez K., Zschock M., Wolter W., Czerny C.P. (2011). Flow cytometric differential cell counts in milk for the evaluation of inflammatory reactions in clinically healthy and subclinically infected bovine mammary glands. J. Dairy Sci..

[11] Wall S.K., Wellnitz O., Bruckmaier R.M., Schwarz D. (2018). Differential somatic cell count in milk before, during, and after lipopolysaccharide- and lipoteichoic-acid-induced mastitis in dairy cows. J. Dairy Sci..

[12] Harmon R.J. (1994). Physiology of mastitis and factors affecting somatic cell counts. J. Dairy Sci..

[13] Damm M., Holm C., Blaabjerg M., Bro M.N., Schwarz D. (2017). Differential somatic cell count-A novel method for routine mastitis screening in the frame of Dairy Herd Improvement testing programs. J. Dairy Sci..

[14] Kirkeby C., Toft N., Schwarz D., Farre M., Nielsen S.S., Zervens L., Hechinger S., Halasa T. (2020). Differential somatic cell count as an additional indicator for intramammary infections in dairy cows. J. Dairy Sci..

[15] Schwarz D., Lipkens Z., Piepers S., De Vliegher S. (2019). Investigation of differential somatic cell count as a potential new supplementary indicator to somatic cell count for identification of intramammary infection in dairy cows at the end of the lactation period. Prev. Vet. Med..

[16] Zecconi A., Dell’Orco F., Vairani D., Rizzi N., Cipolla M., Zanini L. (2020). Differential cell count as a marker for changes of milk composition in cows very low somatic cell counts. Animals.

[17] Goncalves J.L., Lyman R.L., Hockett M., Rodriguez R., dos Santos M.V., Anderson K.L. (2017). Using milk leukocyte differentials for diagnosis of subclinical bovine mastitis. J. Dairy Res..

[18] Rivas A.L., Tadevosyan R., Quimby F.W., Coksaygan T., Lein D.H. (2002). Identification of subpopulations of bovine mammary-gland phagocytes and evaluation of sensitivity and specificity of morphologic and functional indicators of bovine mastitis. Can. J. Vet. Res.-Rev. Can. Rech. Vet..

[19] DVG, G.V.S. (2002). Leitlinien zur Bekämpfung der Mastitis als Bestandsproblem.

[20] Dufour S., Dohoo I.R. (2013). Monitoring herd incidence of intramammaryinfection in lactating cows using repeated longitudinal somatic cellcount measurements. J. Dairy Sci..

[21] Piccinini R., Binda E., Belotti M., Dapra V., Zecconi A. (2007). Evaluation of milk components during whole lactation in healthy quarters. J. Dairy Res..

[22] Schukken Y., Wilson D., Welcome F., Garrison-Tikofsky L., Gonzalez R. (2003). Monitoring udder health and milk quality using somatic cell counts. Vet. Res..

[23] Lindmark-Mansson H., Branning C., Alden G., Paulsson M. (2006). Relationship between somatic cell count, individual leukocyte populations and milk components in bovine udder quarter milk. Int. Dairy J..

[24] Zecconi A., Vairani D., Cipolla M., Rizzi N., Zanini L. (2018). Assessment of Subclinical Mastitis Diagnostic Accuracy by Differential Cell Count in Individual Cow Milk. Ital. J. Anim. Sci..

[25] Yue S., Wang C.Y. (2004). The Mann-Kendall test modified by effective sample size to detect trend in serially correlated hydrological series. Water Resour. Manag..

[26] Zecconi A., Sesana G., Vairani D., Cipolla M., Rizzi N., Zanini L. (2018). Somatic Cell Count as a Decision Tool for Selective Dry Cow Therapy in Italy. Ital. J. Anim. Sci..

[27] Schwarz D., Diesterbeck U.S., Konig S., Brugemann K., Schlez K., Zschock M., Wolter W., Czerny C.P. (2011). Microscopic differential cell counts in milk for the evaluation of inflammatory reactions in clinically healthy and subclinically infected bovine mammary glands. J. Dairy Res..

[28] Stocco G., Summer A., Cipolat-Gotet C., Zanini L., Vairani D., Dadousis D., Zecconi A. (2020). Differential cell count as a novel indicator of milk quality in dairy cows. Animals.

[29] Dosogne H., Vangroenweghe F., Mehrzad J., Massart-Leen A.M., Burvenich C. (2003). Differential leukocyte count method for bovine low somatic cell count milk. J. Dairy Sci..

[30] Leitner G., Chaffer M., Krifucks O., Glickman A., Ezra E., Saran A. (2000). Milk leucocyte populations in heifers free of udder infection. J. Vet. Med. Ser. B-Infect. Dis. Vet. Public Health.

[31] Rainard P., Foucras G., Boichard D., Rupp R. (2018). Invited review: Low milk somatic cell count and susceptibility to mastitis. J. Dairy Sci..

[32] Green L.E., Schukken Y.H., Green M.J. (2006). On distinguishing cause and consequence: Do high somatic cell counts lead to lower milk yield or does high milk yield lead to lower somatic cell count?. Prev. Vet. Med..

[33] Mazzilli M., Zecconi A. (2010). Assessment of epithelial cells’ immune and inflammatory response to Staphylococcus aureus when exposed to a macrolide. J. Dairy Res..

[34] Yang W., Zerbe H., Petzl W., Brunner R.M., Guenther J., Draing C., von Aulocke S., Schuberth H.J., Seyfert H.M. (2008). Bovine TLR2 and TLR4 properly transduce signals from Staphylococcus aureus and E-coli, but S-aureus fails to both activate NF-kappa B in mammary epithelial cells and to quickly induce TNF alpha and interleukin-8 (CXCL8) expression in the udder. Mol. Immunol..

[35] Paudyal S., Pena G., Melendez P., Roman-Muniz I.N., Pinedo P.J. (2018). Relationships among quarter milk leukocyte proportions and cow and quarter-level variables under different intramammary infection statuses. Transl. Anim. Sci..

[36] Cardozo L.L., Neto A.T., Souza G.N., Picinin L.C.A., Felipus N.C., Reche N.L.M., Schmidt F.A., Werncke D., Simon E.E. (2015). Risk factors for the occurrence of new and chronic cases of subclinical mastitis in dairy herds in southern Brazil. J. Dairy Sci..

[37] Zecconi A., Frosi S., Cipolla M., Gusmara C. (2018). Effects of chronic mastitis and its treatment with ketoprofen on the milk ejection curve. J. Dairy Res..

