# Supplementary material for: Factors Affecting the Patterns of Total Amount and Proportions of Leukocytes in Bovine Milk
Source: Animals (Basel). 2020 Jun 6;10(6):992. doi: 10.3390/ani10060992 (PMC7341286; doi:10.3390/ani10060992)
Supplement: Supplementary file 1 [file animals-10-00992-s001.pdf]

animals

MDPI

Supplementary materials

Table S1. Main characteristics of the 12 herds included into the study.

Herd	Samples (N)	Milk yield (Kg/d)	SCC[1] (log_{10}/ml)	DSCC[2] (%)	P+LT[3] (Log_{10} cells)
1	1,267	38.16±8.63	4.66±0.30	55.35±14.50	8.94±0.36
2	4,445	47.10±12.29	4.51±0.32	52.86±15.99	8.87±0.39
3	1,067	35.78±7.81	4.55±0.32	52.51±14.86	8.80±0.39
4	1,476	34.42±8.81	4.66±0.29	56.11±15.32	8.92±0.35
5	1,202	27.56±7.88	4.68±0.27	58.05±13.14	8.85±0.33
6	1,352	32.29±7.68	4.62±0.31	54.96±14.03	8.84±0.37
7	1,168	27.07±6.35	4.86±0.25	64.12±0.65	9.09±0.30
8	860	29.96±7.70	4.65±0.30	57.70±14.55	8.85±0.37
9	1,.379	34.43±9.66	4.061±0.32	56.01±15.34	8.85±0.38
10	1,100	33.47±7.49	4.65±0.32	57.78±14.73	8.91±0.39
11	1,128	31.59±7.72	4.63±0.30	54.32±13.90	8.84±0.36
12	1.485	31.30±8.13	4.60±0.27	54.69±13.74	8.81±0.34

[1]SCC = Somatic Cell Count; [2]DSCC = Differential leukocyte count; [3]P+LT is equal to SCC x milk yield x DSCC (as proportion)

Table S2. Distribution of samples within the two data subsets (healthy and diseased) ranked by parity and SCC.

Status	SCC[1] level	Parity				
		1	2	3	4 or more	Total
healthy	≤50,000	3,323	1,929	959	691	6,902
	≤100,000	4,801	2,948	1,587	1,145	10,481
	≤150,000	5,439	3,379	1,900	1,426	12,144
	≤200,000	5,765	3,642	2,088	1,592	13,087
diseased	>200,000	1,455	1,316	988	1,093	4,852
	>400,000	801	784	629	708	2,922
	>800,000	431	448	361	456	1,696

[1]SCC = Somatic Cell Count

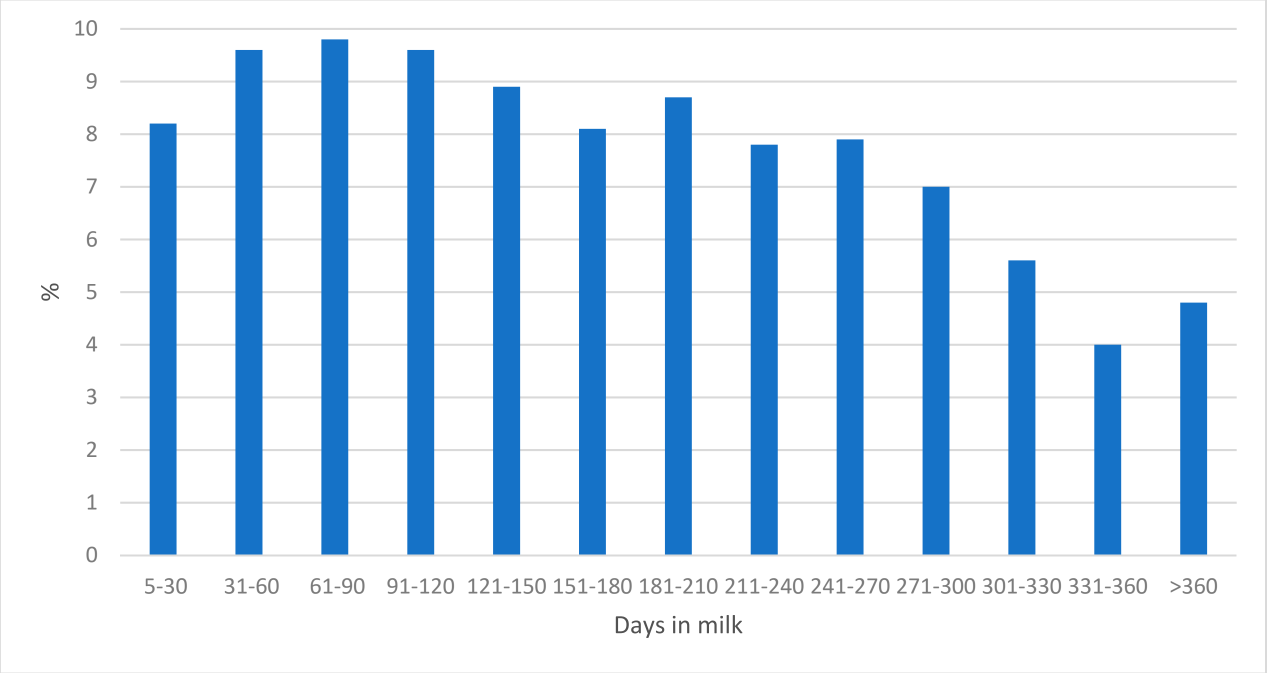

Figure S1. Distribution of samples by days in milk
